# Real‐world outcome of crizotinib for anaplastic lymphoma kinase‐positive lung cancer: Multicenter retrospective analysis in South Korea

**DOI:** 10.1111/1759-7714.15213

**Published:** 2024-01-03

**Authors:** Da Som Jeon, Cheol‐kyu Park, Seung Joon Kim, Chan Kwon Park, Yoon Soo Chang, Chi Young Jung, Sung Yong Lee, Shin‐Yup Lee, Jeong‐Seon Ryu, Jeong Eun Lee, Kye Young Lee, Tae Won Jang, Seung Hun Jang, Seong Hoon Yoon, Sang Hoon Lee, Chang‐min Choi, Hyeong Ryul Kim, Yeon Joo Kim

**Affiliations:** ^1^ Department of Pulmonary and Critical Care Medicine Nowon Eulji Medical Center, University of Eulji Seoul South Korea; ^2^ Department of Pulmonary and Critical Care Medicine Chonnam National University Hwasun hospital, Chonnam National University Jeollanam‐do Republic of Korea; ^3^ Department of Internal Medicine Postech‐Catholic Biomedical Engineering Institute, College of Medicine, The Catholic University of Korea Seoul Republic of Korea; ^4^ Department of Pulmonary and Critical Care Medicine Catholic University of Korean Yeouido Saint Mary's Hospital Seoul Korea; ^5^ Department of Internal Medicine Yonsei University College of Medicine, 8th Floor Annex Building, Yongdong Severance Hospital Seoul Republic of Korea; ^6^ Department of Internal Medicine Daegu Catholic University School of Medicine Daegu Korea; ^7^ Division of Pulmonary, Allergy, and Critical Care Medicine, Department of Internal Medicine, Korea University Guro Hospital, Korea University College of Medicine Seoul Korea; ^8^ Division of Pulmonary and Critical Care Medicine Kyungpook National University Chilgok Hospital Daegu Korea; ^9^ Department of Pulmonary and Critical Care Medicine Inha University Hospital Incheon Republic of Korea; ^10^ Department of Internal Medicine Chungnam National University Hospital Daejeon Republic of Korea; ^11^ Department of Pulmonary Medicine Konkuk University School of Medicine Seoul Republic of Korea; ^12^ Department of Internal Medicine Kosin University Medical College Pusan Korea; ^13^ Department of Pulmonary Allergy and Critical Care Medicine, Hallym University Sacred Heart Hospital Anyang Republic of Korea; ^14^ Department of Internal Medicine School of Medicine, Pusan National University Yangsan Republic of Korea; ^15^ Division of Pulmonology, Institute of Chest Disease, Department of Internal Medicine Yonsei University College of Medicine Seoul Republic of Korea; ^16^ Department of Pulmonary and Critical Care Medicine Asan Medical Centre, University of Ulsan College of Medicine Seoul Republic of Korea; ^17^ Department of Oncology Asan Medical Centre, University of Ulsan College of Medicine Seoul Republic of Korea

**Keywords:** adverse events, anaplastic lymphoma kinase, crizotinib, non‐small cell lung carcinoma, progression‐free survival

## Abstract

**Background:**

About 3%–5% of non‐small cell lung cancer (NSCLC) presents positive anaplastic lymphoma kinase (ALK). Recently, several target agents have been approved as a treatment for ALK‐positive NSCLC. This study aimed to analyze the real‐world efficacy and outcome when administered crizotinib, the first approved target agent for ALK‐positive NSCLC, according to first‐ or late‐line treatment.

**Methods:**

A total of 290 patients with ALK‐positive advanced NSCLC who were treated with crizotinib in 15 institutions in South Korea from January 2009 to December 2018 were enrolled.

**Results:**

The median age of patients was 57.0 years, and 50.3% were male. The median follow‐up duration was 29.3 months. Among them, 113 patients received crizotinib as first‐line therapy. The objective response rate (ORR) was 60.1% (57.0% for first‐line recipients, 61.8% for second−/later‐line). Median (95% CI) progression‐free survival (PFS) was 13.7 (11.6–17.0) months. For first‐line recipients, overall survival (OS) was 26.3 (17.6–35.0) months. No significant difference in ORR, PFS and OS, according to the setting of crizotinib initiation, was observed. In a multivariate Cox regression analysis, old age, male gender, initially metastatic, and number of metastatic organs were associated with poor PFS and OS. The most common adverse events were nausea and vomiting, and severe adverse event leading to dose adjustment was hepatotoxicity.

**Conclusions:**

ORR, PFS, OS, and adverse event profiles were comparable to previous clinical trials. Our findings could aid in the efficient management of ALK‐positive lung cancer patients.

## Graphical abstract

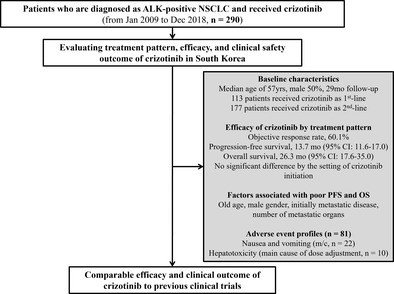

## INTRODUCTION

Lung cancer is the leading cause of cancer‐related deaths worldwide. In South Korea, the age‐standardized mortality rate was 13.4 per 100 000 in 2022, the highest among all site cancers.^1^ Recently, with an increased understanding of the molecular heterogeneity that drives carcinogenesis, non‐small cell lung cancer (NSCLC) is subclassified by the presence of specific oncogenic mutations, and new targeted therapies are already commercialized, proving their superiority to conventional chemotherapy.^2^, ^3^

Anaplastic lymphoma kinase (ALK) is constitutively activated due to the gene rearrangement of echinoderm microtubule‐associated protein like‐4‐ALK (EML‐4‐ALK), which is detected in 3%–5% of NSCLC patients.^4^ Crizotinib is an inhibitor of ALK kinase activity that has been previously reported to achieve higher response rates and a significantly longer median progression‐free survival (PFS) than cytotoxic chemotherapy and was first approved in the various international markets for the standard treatment of patients with metastatic ALK‐positive NSCLC.^5^, ^6^, ^7^ Recently, other target agents such as alectinib and lorlatinib have also been developed, and are being administered to ALK‐positive NSCLC treatment with crizotinib in clinical practice.^8^

Despite the effectiveness of ALK inhibitors (ALKis), patients ultimately develop resistance to therapy.^9^ In addition, because of the toxicity of each ALKis, there are many cases in which dose adjustment is required or the drug needs to be changed.^10^ In this regard, predicting the efficacy and adverse events during crizotinib treatment is important to clinicians. However, there is limited data describing the use of ALKis and their outcomes in real‐world practice settings in Korea.

The objective of the current study was therefore to assess real‐world efficacy of crizotinib in patients with ALK‐positive NSCLC and to identify the factors associated with PFS and overall survival (OS) after crizotinib initiation in regular clinical practice, especially in South Korea.

## METHODS

### Study population and patient selection

The current study only included advanced NSCLC patients treated with crizotinib at 15 institutions in South Korea (Korea Academy of Tuberculosis and Respiratory Diseases [KATRD] Molecular Lung Cancer Study Group) who met all of the following criteria^1^: diagnosed as ALK‐positive (ALK FISH positive) NSCLC between January 1, 2009 and December 31, 2017^2^; 19 years of age or older at the time of diagnosis^3^; initially diagnosed with locally advanced or metastatic NSCLC^4^ and subjects in whom crizotinib was initiated as first‐ or later‐line of therapy for metastatic ALK positive NSCLC. Patients who had pre‐existing or coexisting malignancies in other areas were excluded. Data on the clinical characteristics, previous treatments, and outcomes were retrospectively extracted from the patients' medical records using a secure, web‐based data collection form. All subject data were deidentified and kept anonymous. The study protocol was approved by the Institutional Review Board of each medical center, and informed consent was waived because of the retrospective nature of the study.

### Study variables and endpoints

Baseline demographic characteristics and clinical characteristics such as age, sex, smoking history, histological subtype, metastatic organs (brain, lung‐to‐lung, bone, liver, lymph node, and pleura, etc.) before crizotinib treatment and the presence or absence of previous surgery or irradiation were extracted from each patient's medical record. Treatment patterns of crizotinib including time from diagnosis to crizotinib initiation, last adjusted crizotinib dose, and reason for crizotinib dose change or final discontinuation were assessed and compared by a line of crizotinib treatment (first‐ or second−/later‐line). Adverse events (AEs) related to crizotinib treatment were reported according to the Common Terminology Criteria for Adverse Events, version 4.0.

Objective response rate (ORR) was defined as the proportion of patients achieving the best clinical response to crizotinib of either complete response or partial response (PR), as recorded in the patient's medical record, based on Response Evaluation Criteria in Solid Tumors (RECIST) version 1.1.^11^ PFS was calculated from the starting date of crizotinib treatment to the date of disease progression (PD) confirmed by imaging, death before the initiation of a new therapy, or the last available medical record if censored. OS was measured from the initiation of crizotinib treatment until any cause of death and patients still alive at the time of data collection were censored at the date of data collection.

### Statistical analysis

Analysis variables were summarized and stratified by the setting (first‐line vs .second−/later‐line) in which crizotinib was initiated for the treatment of metastatic NSCLC. Significant differences in descriptive variables between these groups were assessed with the chi‐squared or Fisher's exact tests for qualitative variables and the dtudent's *t*‐test for quantitative variables. The Kaplan–Meier method was used to estimate PFS and OS and survival differences by line of crizotinib were assessed using a nonparametric log‐rank test. We estimated hazard ratios (HRs) and 95% confidence intervals (CIs) using a Cox proportional hazard regression model. Univariate Cox models were applied to select the most promising prognostic variables (threshold *p* = 0.10). A multivariate Cox model was then applied using a backward procedure to adjust for potential confounders. *p* < 0.05 was considered statistically significant for all tests. All statistical analyses were conducted using the IBM SPSS software version 27.0 (IBM Corporation).

## RESULTS

### Clinical characteristics of study population

**FIGURE 1:**
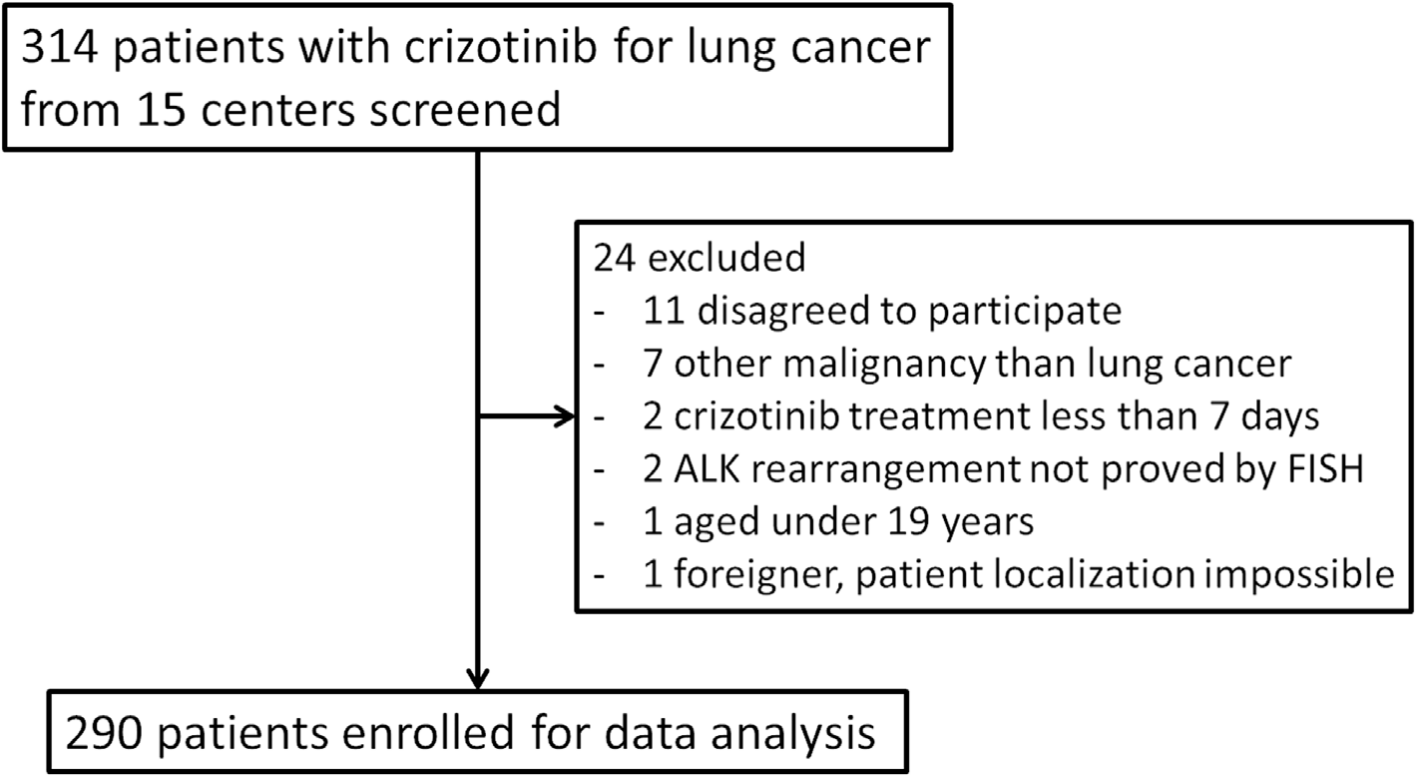
Flow chart of the study. From 314 screened patients from 15 institutions of South Korea, 290 patients were finally enrolled for analysis. ALK, anaplastic lymphoma kinase; FISH, fluorescence in situ hybridization.

A total of 314 patients were initially screened for data collection and analysis. Of these, 290 patients were finally identified for study inclusion (Figure 1). A total of 113 patients received crizotinib as first‐line palliative chemotherapy, while 177 patients had crizotinib therapy as second or later‐line (Table 1). In the overall population, the median (range) age at diagnosis of recurred or metastatic ALK+ NSCLC was 57.0 (20.0–84.0) years, and first‐line recipients were significantly older than second‐ or later‐line recipients (62.0 [20.0–84.0] years vs. 55.0 [26.0–82.0] years). A total of 66 (22.8%) patients had brain metastasis at, or prior to, initiation of crizotinib and first‐line recipients had more frequent brain metastasis compared to second−/later‐line recipients (32.7% vs. 16.4%). Patients who received crizotinib as second‐ or later‐line therapy had a larger proportion of recurrent NSCLC compared to the first‐line crizotinib recipient group (22.6% vs. 8.0%). The second−/later‐line recipient group therefore had 29.4% and 41.8% of patients who underwent surgery and radiotherapy, respectively, which was a significantly larger number than the other group. A total of 67 (23.1%) of total patients had more than two metastatic organs at the time of diagnosis, which did not differ by line of crizotinib therapy. A total of 140 (48.3%) of total subjects were alive at the time of record abstraction, with the proportion of living patients appearing to be lower in the first‐line group. The median total observational duration, from crizotinib initiation until the last available medical record, was 29.4 months, while the first‐line recipient group had a significantly shorter duration of follow‐up (19.2 months) than the other group (38.9 months).

**TABLE 1:** Demographics and clinical characteristics.

Characteristics	All patients (*n* = 290)	Setting of crizotinib initiation		
		First‐line	Second‐ or later‐line	*p*‐value
		(*n* = 113)	(*n* = 177)	
Age (years) at diagnosis	57.0 (20.0–84.0)	62.0 (20.0–84.0)	55.0 (26.0–82.0)	0.004
Median (range)^a^				
Male	146 (50.3)	60 (53.1)	86 (48.6)	0.454
Smoking status at diagnosis^a^				0.231
Current smoker	54 (18.6)	27 (23.9)	27 (15.3)	
Former smoker	67 (23.1)	22 (19.5)	45 (25.4)	
Never smoked	163 (56.2)	61 (54.0)	102 (57.6)	
Unknown	6 (2.1)	3 (2.7)	3 (1.7)	
Palliative reason				0.001
Recurred	49 (16.9)	9 (8.0)	40 (22.6)	
Initially advanced or metastatic	241 (83.1)	104 (92.0)	137 (77.4)	
Histological type				0.234
Adenocarcinoma	280 (96.6)	108 (95.6)	172 (97.2)	
Squamous cell carcinoma	3 (1.0)	1 (0.9)	2 (1.1)	
Adenosquamous carcinoma	1 (0.3)	0 (0.0)	1 (0.6)	
Sarcomatid carcinoma	1 (0.3)	0 (0.0)	1 (0.6)	
NSCLC	3 (1.0)	3 (2.7)	0 (0.0)	
Other	2 (0.7)	1 (0.9)	1 (0.6)	
Mutation other than ALK				
EGFR (+)	8 (2.8)	1 (0.9)	7 (4.0)	0.237
KRAS (+)	2 (0.7)	0 (0.0)	2 (1.1)	0.002
Brain metastasis at diagnosis	66 (22.8)	37 (32.7)	29 (16.4)	0.001
Number of metastatic organs ≥3	67 (23.1)	32 (28.3)	35 (19.8)	0.092
Vital status at medical record abstraction				0.265
Alive	140 (48.3)	48 (42.5)	92 (52.0)	
Deceased	93 (32.0)	38 (33.6)	55 (31.1)	
Transfer	31 (10.7)	13 (11.5)	18 (10.2)	
Follow‐up loss	26 (9.0)	14 (12.4)	13 (11.5)	
Other cancer‐directed therapies administered prior to crizotinib initiation				
Surgery	63 (21.7)	15 (13.3)	48 (27.1)	0.005
Radiotherapy	69 (23.8)	13 (11.5)	56 (31.6)	0.001
Use of ALKis after crizotinib failure	111 (38.3)	35 (31.0)	76 (42.9)	0.041
Duration (months) of observation, from crizotinib initiation until last available medical record, median (95% CI)	29.3 (26.9–31.7)	19.2 (17.8–20.6)	38.9 (34.1–43.7)	<0.001
Crizotinib response				0.014
CR	3 (1.0)	1 (0.9)	2 (1.1)	
PR	152 (52.4)	52 (46.0)	100 (56.5)	
SD	84 (29.0)	30 (26.5)	54 (30.5)	
PD	19 (6.6)	10 (8.8)	9 (5.1)	
Not evaluable	32 (11.0)	20 (17.7)	12 (6.8)	
ORR, %	60.1	57.0	61.8	0.447

*Note*: Values are presented as mean (standard deviation) or number (%) unless otherwise indicated.

Abbreviations: ALK, anaplastic lymphoma kinase; ALKis, ALK inhibitors; CI, confidence interval; CNS, central nervous system; CR, complete remission; EGFR, epidermal growth factor receptor; NSCLC, non‐small cell lung cancer; ORR, objective response rate; PD, progressive disease; PR, partial response; SD, stable disease; VEGF, vascular endothelial growth factor.

### Crizotinib efficacy and analysis of survival with crizotinib

**FIGURE 2:**
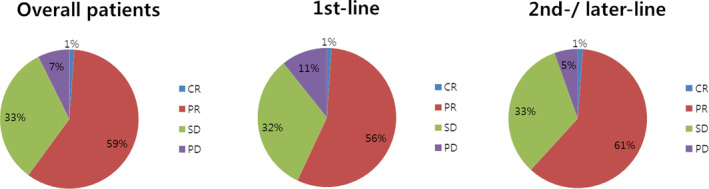
Best clinical response during crizotinib treatment. CR, complete response; PD, progression of disease; PR, partial response; SD, stable disease.

**FIGURE 3:**
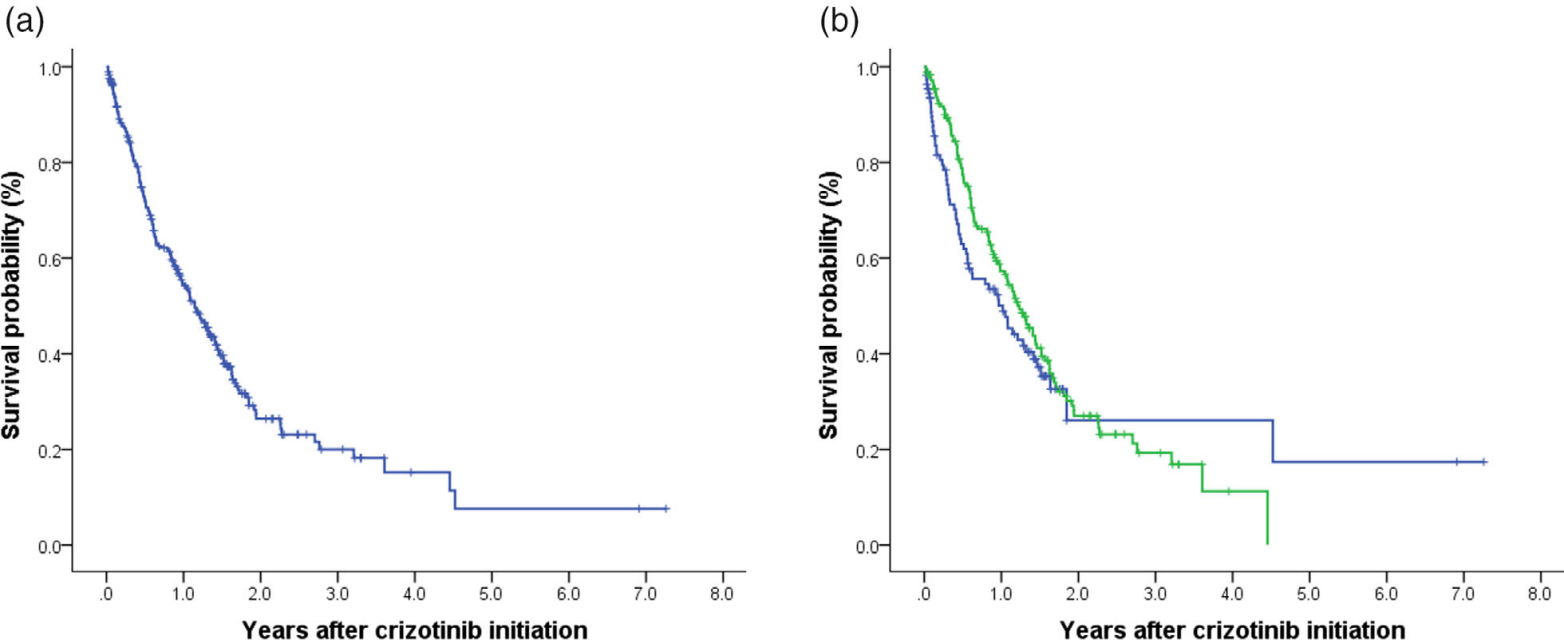
Progression‐free survival from crizotinib initiation. Progression‐free survival is shown in (a) overall population and (b) by lines of crizotinib treatment. Blue line represents first‐line treatment, green line represents second−/later‐line treatment of crizotinib.

**FIGURE 4:**
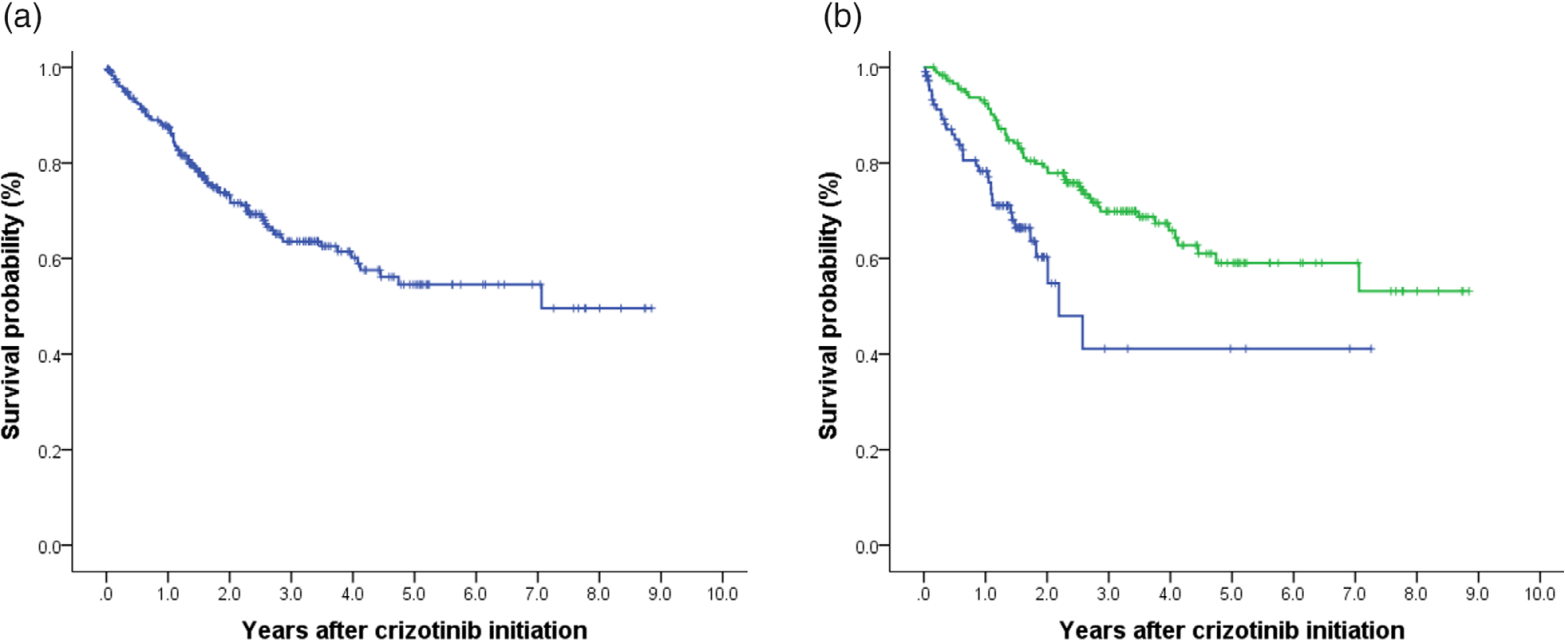
Overall survival from crizotinib initiation. Overall survival is shown in (a) overall population and (b) by lines of crizotinib treatment. Blue line represents first‐line treatment, green line represents second−/later‐line treatment of crizotinib.

The median number of months to crizotinib initiation after initial metastatic NSCLC diagnosis was 5.9 months in the overall population. In the overall patients, the ORR for crizotinib treatment was 60.1%. Patients in whom crizotinib was initiated as first‐line treatment had no significant superiority in response to the drug to the second‐/later‐line group of patients. PR during crizotinib treatment was the most common best clinical response recorded (Table 1, Figure 2). Stable disease (SD) was recorded as best response for 29.0% of the patients and 6.6% experienced PD as their best clinical response during crizotinib treatment. Median PFS (95% CI) from crizotinib initiation was 13.7 (11.4–16.1) months (Table 2, Figure 3a); by setting of crizotinib initiation, median PFS estimates were 11.6 (6.7–16.5) for first‐line and 14.6 (11.9–17.2) for second−/later‐line initiators, respectively (*p* = 0.282, Figure 3b). From crizotinib initiation, OS did not reach to median for the overall cohort. For patients in whom crizotinib was initiated as first‐line treatment, the median (95% CI) OS was 26.3 (17.6–35.0) months, while OS for second−/later‐line crizotinib initiator did not reach to median (*p* = 0.109, Table 2, Figure 4).

**TABLE 2:** Kaplan–Meier point estimates of progression‐free and overall survival.

	All patients (*n* = 290)	Setting of crizotinib initiation		
		First‐line	Second‐ or later‐line	*p*‐value
		(*n* = 113)	(*n* = 177)	
Progression‐free survival				0.282
Mean (SE)	21.7 (2.3)	25.7 (4.9)	19.5 (1.5)	
Median (95% CI)	13.7 (11.4–16.1)	11.6 (6.7–16.5)	14.6 (11.9–17.2)	
Q1, Q3	5, 27	4, 54	7, 27	
Overall survival				0.109
Mean (SE)	66.5 (3.3)	43.8 (6.4)	72.3 (3.7)	
Median (95% CI)	NR	26.3 (17.6–35.0)	NR	
Q1, Q3	19, NR	13, NR	31, NR	

Abbreviation: CI, confidence interval. NR, not reached

### Predicting factors associated to PFS and OS

Univariate and multivariate Cox regression analyses for PFS and OS after crizotinib initiation were performed (Tables 3 and 4). Univariate Cox analysis showed old age, male gender, smoker, initially metastatic disease (not recurred disease), ≥3 metastatic organs, and presence of brain metastasis at the time of crizotinib initiation significantly affected shorter PFS, while old age, male gender, initially metastatic disease, number of metastasis and baseline brain metastasis were the statistically significant factor associated with shorter OS. Multivariate Cox analysis revealed that old age, male gender, initially metastatic, and number of metastatic organs were associated with PFS and OS.

**TABLE 3:** Univariate and multivariate Cox regression analysis of progression‐free survival with crizotinib.

	Progression‐free survival			
	Univariate		Multivariate	
Variables	HR (95% CI)	*p*‐value	HR (95% CI)	*p*‐value
Age	1.016 (1.003–1.029)	0.012	1.020 (1.007–1.033)	0.002
Male	1.498 (1.128–1.988)	0.005	1.671 (1.112–2.512)	0.013
Smoking history				
Nonsmoker	1.000		1.000	
Current smoker	1.824 (1.271–2.618)	0.001	0.728 (0.458–1.156)	0.178
Ex‐smoker	1.083 (0.758–1.548)	0.660	1.318 (0.821–2.117)	0.253
Palliative reason				
Recurred	1.000		1.000	
Initially metastatic	2.244 (1.438–3.501)	<0.001	2.106 (1.333–3.326)	0.001
No. of meta ≥3	2.385 (1.753–3.246)	<0.001	2.124 (1.548–2.914)	<0.001
Baseline brain metastasis	1.914 (1.400–2.615)	<0.001	1.330 (0.937–1.886)	0.111
Line of crizotinib				
First‐line	1.000			
Second‐ or later‐line	0.816 (0.608–1.095)	0.175		
Response to crizotinib				
Responder (CR + PR)	1.000			
No responder (SD + PD)	1.133 (0.836–1.536)	0.420		

*Note*: Values are presented as hazards ratio (HR) or 95% confidence interval (CI) unless otherwise indicated.

Abbreviations: CI, confidence interval; CR, complete response; N/A, not applicable; ORR, objective response rate; PD, progressive disease; PR, partial response; SD, stable disease.

**TABLE 4:** Univariate and multivariate Cox regression analysis of overall survival with crizotinib.

	Overall survival			
	Univariate		Multivariate	
Variables	HR (95% CI)	*p*‐value	HR (95% CI)	*p*‐value
Age	1.023 (1.005–1.041)	0.011	1.018 (1.001–1.036)	0.042
Male	1.624 (1.075–2.454)	0.021	1.790 (1.182–2.710)	0.006
Smoking history				
Nonsmoker	1.000			
Current smoker	1.513 (0.890–2.574)	0.126		
Ex‐smoker	1.150 (0.695–1.904)	0.586		
Palliative reason				
Recurred	1.000		1.000	
Initially metastatic	1.545 (1.075–2.221)	0.019	2.266 (1.179–4.358)	0.014
No. of meta ≥3	2.395 (1.571–3.652)	<0.001	2.136 (1.376–3.314)	0.001
Baseline brain metastasis	1.693 (1.091–2.628)	0.019		
Line of crizotinib				
First‐line	1.000			
Second‐ or later‐line	0.705 (0.463–1.071)	0.102		
Use of ALKis after crizotinib failure	0.525 (0.340–0.810)	0.004	0.479 (0.306–0.751)	0.001
Response to crizotinib				
Responder (CR + PR)	1.000			
No responder (SD + PD)	1.087 (0.703–1.682)	0.707		

*Note*: Values are presented as hazards ratio (HR) or 95% confidence interval (CI) unless otherwise indicated.

Abbreviations: ALKis, anaplastic lymphoma kinase inhibitors; CI, confidence interval; CR, complete response; N/A, not applicable; ORR, objective response rate; PD, progressive disease; PR, partial response; SD, stable disease.

### Safety profile of crizotinib

Adverse events leading to dose reduction or treatment discontinuation during the administration of crizotinib are shown in Table 5. Of 290 patients, 81 (27.9%) had one or more AEs after the administration of crizotinib. The most common AEs were gastrointestinal related such as nausea and vomiting (3.1% and 4.5%, respectively). The following AEs were hepatotoxicity (3.4%), pneumonitis (2.4%) and pneumonia (2.1%). A total of 46 patients (15.9%) required dose reduction or discontinuation of crizotinib, the most common cause of dose adjustment was hepatotoxicity, followed by neutropenia, vomiting, and nausea. The most commonly adjusted dosage of crizotinib was 200 mg b.i.d. (42.9% of patients, Table S1). Dose adjustment of crizotinib was more frequent in the second‐ or later‐line crizotinib recipient group (20.9% vs. 10.6%, Table S1).

**TABLE 5:** Incidence of treatment‐related adverse events and leading to dose reduction or discontinuation.

	All patients (*n* = 290)
Adverse events	81 (27.9)
Nausea	9 (3.1)
Vomiting	13 (4.5)
Neutropenia	9 (3.1)
Hepatotoxicity	10 (3.4)
Heartburn	1 (0.3)
General malaise	1 (0.3)
Anorexia	5 (1.7)
Complicated kidney cyst	5 (1.7)
Leg edema	3 (1.0)
Pneumonitis	7 (2.4)
Pneumonia	6 (2.1)
Skin rash	3 (1.0)
Other intolerability reported by patient	31 (10.7)
Severe adverse events leading to dose adjustment or discontinuation	46 (15.9)
Hepatotoxicity	9 (3.1)
Neutropenia	8 (2.8)
Vomiting	6 (2.1)
Nausea	4 (1.4)
Pneumonitis	3 (1.0)
Complicated kidney cyst	3 (1.0)
Anorexia	2 (0.7)
Leg edema	1 (0.3)
Heartburn	1 (0.3)
Others	9 (3.1)

*Note*: Data are expressed as the number of patients (%).

## DISCUSSION

To the best of our knowledge, this is the first study exploring real‐world data of patients with ALK‐positive NSCLC treated with crizotinib in South Korea. In this study, the overall response rate to crizotinib was 60.1%, and median PFS was 13.7 months. OS did not reach to median for the overall cohort, and around 15% of patients required dose reduction because of AE. Data describing the use of crizotinib and its outcomes among ALK‐positive metastatic NSCLC patients in real‐world practice settings are evolving, but the need for Asian data still exists.

The therapeutic effects of crizotinib in advanced ALK‐positive NSCLC have been proven through PROFILE 1014 and PROFILE 1029 studies.^7^, ^12^, ^13^ In the PROFILE 1014 study, the overall response rate of crizotinib was 74%.^7^ A meta‐analysis reported that the ORR of crizotinib was 65%.^14^ In this study, first‐line crizotinib showed 74% ORR in comparison with second‐line crizotinib of 65%; however, there was no statistical significance between the two groups.^14^ In our study, ORR to crizotinib was 60.1% for the overall study sample, 57.0% for first‐line crizotinib initiators, and 61.8% for second−/later‐line initiators. ORR in our study was slightly lower compared to other studies. This might be associated with the fact that our study population contained a larger proportion of recurrent NSCLC patients who had undergone surgery or concurrent chemoradiotherapy, which could alter tumor response to crizotinib. Also, first‐line crizotinib initiators in the current study included an older population and frequent brain metastasis, which might affect the response to crizotinib. Furthermore, the time to crizotinib initiation from the diagnosis being longer than in other studies might be another reason for the lower response.

Median PFS for crizotinib recipients was 13.7 months overall, 11.6 months for first‐line, and 14.6 months for second−/later‐line; however, it was numerically longer than other studies, including 10.9 months for the treatment‐naïve patients reported by Solomon et al.,^7^ 7.7 and 6.8 months for second‐line recipients estimated in Western Countries.^6^, ^15^ In Asian population analysis of J‐ALEX study shows 10.2 months of PFS.^16^ This superiority in PFS in the current study might infer that Korean patients with ALK‐positive NSCLC may receive a longer effect of crizotinib than Western patients, due to different resistance‐acquiring mechanisms or other traits of ALK‐positive NSCLC, although they show a relatively low response rate to crizotinib.

OS was also consistent and appeared to be a little better in the present study compared to previous studies. Median OS from crizotinib initiation in the first‐line setting was not reached in the final analysis of the phase III PROFILE 1014 trial^12^ and Davis et al.^17^ reported 23.4 months of median OS for first‐line recipients, which appears to be generally in line with our data of 26.3 months. As for second−/later‐line therapy, Shaw et al. reported a median OS of 21.7 months for second‐line crizotinib recipients from crizotinib initiation^6^ and 49.5 months for the 145 patients who received at least one ALK inhibitor (crizotinib as a first subsequent treatment for 144 patients and ceritinib for one patient) in any line of subsequent treatment after conventional chemotherapy.^12^ Davis et al.^17^ reported nearly 2 years as median OS for second‐line crizotinib initiators, while our data revealed OS had not reached to median for second−/later‐line crizotinib recipients. The median OS of crizotinib in the current study does not appear to be inferior compared to previous studies. There were also no significant differences by lines of crizotinib treatment in the study by Davis et al.^17^ which was also the result of our study. Recently, the final OS analysis of crizotinib in the ALEX trial was revealed; 57.4 months in stage III/IV ALK‐positive NSCLC. Further research is required on survival data of next‐generation TKIs as well as crizotinib in the near future.

We also analyzed factors that can affect PFS and OS. Old age, male gender, initially metastatic, and number of metastatic organs ≥3 were associated with both poor PFS and OS. This result is comparable to a recent study by Ock et al.,^18^ which identified performance status more than ECOG ≥2, ≥3 metastatic organs at the time of diagnosis, and no response to crizotinib as factors affecting shorter PFS and OS. The study by Ock et al.^18^ was a retrospective cohort study reviewing the patients enrolled in PROFILE 1001, 1005, 1007, and 1014.^6^, ^7^ They developed a model consisting of three predicting factors and validated the model in two validation cohorts to determine if it could make a distinction in prognosis by score. Our data differs with the data by Ock et al. in that the response to crizotinib was not significantly associated with PFS or OS of crizotinib. Rather, male gender and initially metastatic disease status independently affected OS after initiation of chemotherapy. These differences may come from the disparities between the controlled cohort and the unselected real‐world setting population. Further study is needed to identify the validity of the prediction model proposed by Ock et al.^18^

The most common cause for dose adjustment or discontinuation in our study was vomiting (4.5%), followed by hepatotoxicity (3.4%). A total of 84.1% of patients did not require dose adjustment. As in the retrospective study by Davis et al.,^17^ this study assessed the reasons for final crizotinib discontinuation as well as dose changes of crizotinib during the treatment course. Although categories of reason for discontinuation and dose change of crizotinib are not fully comparable with the study by Davis et al., PD was the most commonly cited reason for crizotinib discontinuation in both studies, and more than 80% of patients experienced no change in crizotinib dose during the therapy, which combined about 4% discontinuation rate due to toxicities, implying that there was relatively favorable tolerability of the treatment in both study patients.

Our study had some limitations. First, the current study was retrospective, and the exact performance status could not be assessed from medical records. Second, because this study used real‐world data, it was not as able to control all variables as a randomized controlled trial would have been. However, it was a multicenter retrospective cohort study that could assess real‐world outcomes of crizotinib outside the highly controlled environment of randomized trials. Third, in retrospective studies, response criteria are not dictated by a protocol, and assessments (such as imaging studies) may not be done on a uniform schedule. Therefore, results regarding this endpoint may not be directly comparable to those observed in clinical trials. Finally, due to the 2‐year interval of reimbursement of crizotinib as a second‐ and first‐line chemotherapeutic agent by the Korean FDA, follow‐up duration differs by line of crizotinib treatment, which limits the exact comparison between the outcome of crizotinib between the first‐line initiator and second−/later‐line initiator group. However, the extremely long duration of observation and comparative response rate, PFS, and OS make our results rather reasonable and suggest a further beneficial role of treatment with crizotinib in Korea, which might be related to the unique characteristics of Asian ethnicity.

Despite these limitations, this study provides meaningful information on the use and outcomes of crizotinib in a real‐world population of ALK‐positive metastatic NSCLC patients treated with crizotinib, by conducting a multicenter retrospective cohort in South Korea.

This was a large cohort study that demonstrated the efficacy and favorable outcome of crizotinib in ALK‐positive NSCLC patients in South Korea. Nowadays, crizotinib is used in various molecular mutations such as ROS‐1 and C‐MET as well as ALK‐positive NSCLC, so this study will help clinicians understand efficacy and AEs. Further research on various target agents is needed in South Korea in the near future.

## AUTHOR CONTRIBUTIONS

Hyeong Ryul Kim takes full responsibility for the content of this manuscript, including the data and analysis. Yeon Joo Kim and Hyeong Ryul Kim participated in the development of the study design, interpretation of the data, and critical review of this manuscript. Yeon Joo Kim and Da Som Jeon wrote the first draft of the manuscript. We thank all other study authors for contributing equally to the decision to submit this manuscript for publication.

## CONFLICT OF INTEREST STATEMENT

This study was supported by a grant (2017‐689 to HRK) from the Asan Institute for Life Sciences, Seoul, Korea. The authors have no conflicts of interest to declare otherwise.

## Supporting information


**TABLE S1.** Treatment pattern of crizotinib in study patients.
**TABLE S2.** Demographics according to groups that received crizotinib for recurrence after surgery or at the initial metastatic disease.
**FIGURE S1.** Progression‐free survival (a) and overall survival (b) according to groups that received crizotinib for recurrence after surgery or at the initial metastatic disease.
