# Supplementary material for: Real‐world outcome of crizotinib for anaplastic lymphoma kinase‐positive lung cancer: Multicenter retrospective analysis in South Korea
Source: Thorac Cancer. 2024 Jan 3;15(6):448–57. doi: 10.1111/1759-7714.15213 (PMC10883859; doi:10.1111/1759-7714.15213)
Supplement: Supplementary file 1 — TABLE S1. Treatment pattern of crizotinib in study patients. TABLE S2. Demographics according to groups that received crizotinib for recurrence after surgery or at the initial metastatic disease. FIGURE S1. Progression‐free survival (a) and overall survival (b) according to groups that received crizotinib for recurrence after surgery or at the initial metastatic disease. [file TCA-15-448-s001.docx]

**Supplementary Table 1. Treatment pattern of Crizotinib in study patients**

|  | **All patients** | **Setting of Crizotinib Initiation** | | |
| --- | --- | --- | --- | --- |
|  | (n = 290) | First-Line  (n = 113) | Second- or Later-Line  (n = 177) | P value |
| **Times (months) from initial diagnosis to crizotinib initiation** |  |  |  | <0.001 |
| Mean (SD) | 9.6 (0.9) | 1.4 (0.3) | 14.9 (1.2) |  |
| Median (95% CI) | 2.9 (1.6–4.2) | 0.7 (0.6–0.8) | 9.4 (7.2–11.6) |  |
| Range (minimum, maximum) | 0.0, 101.1 | 0.0, 25.9 | 0.0, 101.1 |  |
| **Last adjusted daily dose of crizotinib prescribed** |  |  |  | 0.469 |
| 200 mg b.i.d. | 22 (44.9) | 7 (58.3) | 15 (40.5) |  |
| 250 mg b.i.d. | 9 (18.4) | 3 (25.0) | 6 (16.2) |  |
| 200 mg q.d. | 3 (6.1) | 0 (0.0) | 3 (8.1) |  |
| 250 mg q.d. | 15 (30.6) | 2 (16.7) | 13 (35.1) |  |
| **Crizotinib dose changes ≥ 1** | 49 (16.9) | 12 (10.6) | 37 (20.9) | 0.023 |
| **Duration (months) of crizotinib treatment, from initiation to last observed dose, median (95% CI)** | 10.2 (7.6–12.9) | 6.8 (4.9–8.6) | 12.1 (9.7–14.6) | 0.166 |
| **Reason(s) for final discontinuation of crizotinib** |  |  |  | 0.815 |
| Ongoing crizotinib treatment | 69 (23.8) | 28 (24.8) | 41 (23.2) |  |
| Disease progression | 160 (55.2) | 59 (52.2) | 101 (57.1) |  |
| Treatment-related toxicity or side effects | 18 (6.2) | 6 (5.3) | 12 (6.8) |  |
| Patient request | 10 (3.4) | 5 (2.8) | 5 (4.4) |  |
| Follow-up loss | 11 (3.8) | 5 (2.8) | 6 (5.3) |  |
| Transfer | 15 (5.2) | 7 (6.2) | 8 (4.5) |  |
| Death | 7 (2.4) | 2 (1.8) | 5 (2.8) |  |

Values are presented as mean (standard deviation) or number (%) unless otherwise indicated. CI = confidence interval.

**Supplementary Table 2.** Demographics according to groups that received crizotinib for recurrence after surgery or at the initial metastatic disease

|  | **Recurrence after surgery** | **Initial metastatic disease** | **P value** |
| --- | --- | --- | --- |
|  | (n = 43) | (n = 215) |  |
| Male | 22 (51.2) | 110 (51.2) | 0.999 |
| Age | 57.0 (48.0–64.0) | 58.0 (49.0–68.0) | 0.749 |
| Never-smoker | 24 (55.8) | 121 (56.3) | 0.723 |
| Metastasis > 3  Brain metastasis  Alimta treatment before administration of crizotinib  Progression-free survival (months)  Overall survival (months) | 2 (4.7)  13 (30.2)  28 (65.1)  30.9 (22.6–39.1)  64.3 (50.7–77.8) | 62 (28.8)  71 (33.0)  129 (60.0)  20.2 (16.3–24.1)  41.7 (34.9–48.4) | <0.001  0.051  0.530  <0.001  <0.001 |

**Supplementary Figure 1.** Progression free survival (A) and overall survival (B) according to groups that received crizotinib for recurrence after surgery or at the initial metastatic disease.

**
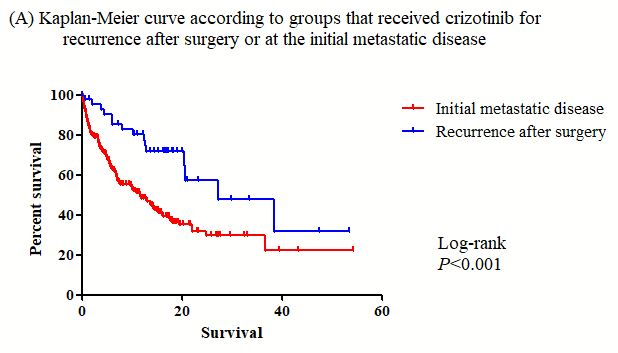

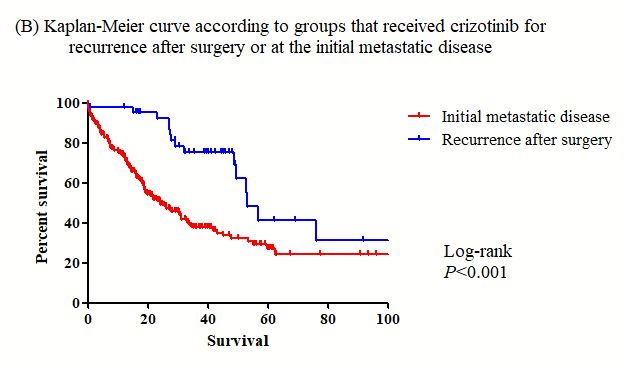
**
